# A call for action: Improve reporting of research studies to increase the scientific basis for regulatory decision‐making

**DOI:** 10.1002/jat.3578

**Published:** 2018-01-03

**Authors:** Marlene Ågerstrand, Sofie Christiansen, Annika Hanberg, Christina Rudén, Lars Andersson, Sjur Andersen, Henrik Appelgren, Christine Bjørge, Ian Henning Clausen, Dag Markus Eide, Nanna B. Hartmann, Trine Husøy, Halldór Pálmar Halldórsson, Marianne van der Hagen, Ellen Ingre‐Khans, Adam David Lillicrap, Vibe Meister Beltoft, Anna‐Karin Mörk, Mari Murtomaa‐Hautala, Elsa Nielsen, Kristín Ólafsdóttir, Jaana Palomäki, Hinni Papponen, Emilie Marie Reiler, Helene Stockmann‐Juvala, Tiina Suutari, Henrik Tyle, Anna Beronius

**Affiliations:** ^1^ Department of Environmental Science and Analytical Chemistry Stockholm University Sweden; ^2^ Division of Diet, Disease Prevention and Toxicology, National Food Institute Technical University of Denmark Kgs. Lyngby Denmark; ^3^ Institute of Environmental Medicine Karolinska Institutet Sweden; ^4^ Swedish Chemicals Agency Sweden; ^5^ Norwegian Environment Agency Trondheim Norway; ^6^ Danish Environmental Protection Agency Copenhagen Denmark; ^7^ Department of Toxicology and Risk assessment Norwegian Institute of Public Health Oslo Norway; ^8^ Department of Environmental Engineering Technical University of Denmark Kgs. Lyngby Denmark; ^9^ University of Iceland's Research Centre in Suðurnes Suðurnes Iceland; ^10^ Ecotoxicology and Risk Assessment Norwegian Institute for Water Research Oslo Norway; ^11^ Division for Risk Assessment and Nutrition, National Food Institute Technical University of Denmark Kgs. Lyngby Denmark; ^12^ Division of Environmental Permits Regional State Administrative Agency for Northern Finland Oulu Finland; ^13^ Department of Pharmacology and Toxicology University of Iceland Iceland; ^14^ Finnish Safety and Chemicals Agency Helsinki Finland; ^15^ Work Environment Finnish Institute of Occupational Health Helsinki Finland

## Abstract

This is a call for action to scientific journals to introduce reporting requirements for toxicity and ecotoxicity studies. Such reporting requirements will support the use of peer‐reviewed research studies in regulatory decision‐making. Moreover, this could improve the reliability and reproducibility of published studies in general and make better use of the resources spent in research.

Toxicity and ecotoxicity studies published in scientific journals can have an impact outside the scientific community by contributing to decision‐making processes in society. As scientific journals are able to communicate efficiently with individual researchers via their guidelines for authors and review processes, their actions are crucial in ensuring adequate reporting of research studies. Society is increasingly asking science for decision support in both direct and indirect ways, and there is consequently a potential for greater collaboration between the scientific community and the regulatory sector. We therefore urge scientific journals to improve the reporting of toxicity and ecotoxicity studies by introducing reporting requirements. Modern technology means much of this work can be automated, for example by asking authors to respond to the reporting requirements before completing the submission of a new paper. This will make it possible for journals to identify studies with inadequate reporting early in the publication process, and will simplify the peer review process.

Reporting recommendations, suitable for toxicity and ecotoxicity studies, are already publicly available via the reporting and evaluation tool SciRAP (http://www.scirap.org) (Beronius, Molander, Rudén, & Hanberg, 2014; Moermond, Kase, Korkaric, & Ågerstrand, 2016). These reporting recommendations are based on the internationally harmonized Organisation for Economic Co‐operation and Development test guidelines to ensure a comprehensive cover of relevant aspects, but also further developed in collaboration with scientists, risk assessors and regulators (Beronius, Ågerstrand, Rudén, & Hanberg, 2017; Kase, Korkaric, Werner, & Ågerstrand, 2016). The reporting recommendations concern aspects such as description of the endpoint, test substance, vehicle, test organism or animal model, test environment, housing conditions (for in vivo toxicity studies), exposure conditions, results and statistics. Adequate reporting of these aspects is crucial for the understanding of a study's strengths and limitations, and thereby it's potential regulatory use. Systematic reporting recommendations have already been implemented in several other research areas, e.g., *Nature*’s reporting checklist for life sciences articles, the ARRIVE guideline for in vivo toxicity studies, the STROBE statement in the field of epidemiology and the MIAME reporting standard for microarray experiments (Brazma et al., 2001; Kilkenny, Browne, Cuthill, Emerson, & Altman, 2010; *Nature*, 2013; Vandenbroucke, von Elm, Altman, Gøtzsche, & Mulrow, 2007).

We also encourage the regulatory sector to take action. Guidance for hazard and risk assessment of chemicals generally requires or recommends that all relevant data should be taken into account. Non‐standard studies can contribute with critical data and fill information gaps. Such an example is identifying and assessing endocrine disrupting chemicals, as research studies using novel methods and endpoints may be more sensitive and relevant for assessing endocrine‐related outcomes (EFSA Scientific Committee, 2013; Kortenkamp et al., 2011). Similar considerations apply to nanomaterials as they are known to behave differently in ecotoxicity tests compared to conventional chemicals, for which most test guidelines were developed (Hartmann, Ågerstrand, Lützhøft, & Baun, 2017), as well as veterinary and human medicines as they may have specific effects on non‐target organisms through interactions with drug targets (Lillicrap, Macken, & Thomas, 2015; Ågerstrand et al., 2015). In spite of this, studies conducted in accordance with standardized test guidelines, such as the Organisation for Economic Co‐operation and Development test guidelines, and Good Laboratory Practices are sometimes by default attributed higher reliability than (academic) research studies and preferred for regulatory hazard and risk assessments (Kase et al., 2016). This example shows the importance of adequate reporting so that peer‐reviewed research studies can be better understood and included in regulatory assessments. From a European perspective, the European Commission, the European Chemicals Agency and the European Food Safety Authority are some of the stakeholders with the potential to influence the scientific community to improve the reporting of studies to meet regulatory requirements.

For some time, researchers, decision makers and scientific journals have discussed the issue of insufficient reporting of peer‐reviewed studies and the consequences for the confidence in individual study results and in science in general (Glasziou et al., 2014; Hanson et al., 2017). An insufficiently reported study is not necessarily a study of low scientific quality. However, it is often perceived as such if information to understand the results and evaluate their validity is missing. This is unfortunate, as it undermines the trust in science and, importantly, hampers the integration of peer‐reviewed research studies into the scientific basis for decision‐making processes.

Within the field of chemical assessment, there are several examples of controversies caused partly by disagreement between experts regarding the reliability and relevance of studies published in the peer‐reviewed literature. Examples include, in the USA, the widely used herbicide atrazine, and the brominated flame retardant decaBDE, which now is proposed to be regulated both in Europe and in the Stockholm Convention on Persistent Organic Pollutants (Alcock, MacGillivray, & Busby, 2011; Boone et al., 2014). Insufficient reporting of design, conduct and results can partly explain the reduced credibility of individual studies. Consequently, regulators and risk assessors are struggling with how to use such studies as part of the scientific basis for chemicals regulation. Unfortunately, peer‐reviewed studies can therefore be ineligible for regulatory use even though they have the potential to provide critical data and fill important information gaps (Kortenkamp et al., 2011; Zoeller et al., 2012). Despite this being a concrete and well‐defined problem, current measures for how to deal with it remain insufficient.

It is our experience that academic researchers, in general, are not well aware of regulatory processes and the requirements placed on scientific data that are used in hazard and risk assessment of chemicals (Ågerstrand et al., 2017). This low awareness could result from a low interest in regulatory aspects and regulatory use of studies. However, this is not in line with many academic researchers’ aspiration of preserving and protecting the environment and human health. Therefore, a possible alternative explanation could be that this is the result of the scientific community and the regulatory sector working isolated from each other, or without sufficient interaction, for a long time and thereby developing somewhat incompatible work cultures.

It could be argued that it is not the role of the scientific community to deliver data for chemicals regulation and decision‐making, and that this responsibility belongs to industry and regulatory agencies. It could also be considered that the primary purpose of studies from the peer‐reviewed literature is to contribute to knowledge and innovation. Both are indeed true. However, current chemical regulations such as the REACH (EC 1907/2006), state that all relevant data should be considered when conducting hazard and risk assessment of chemicals. Studies should be evaluated for their adequacy to a particular assessment, irrespective of who performed the study and for what purpose (European Chemicals Agency, 2011). Furthermore, the aspects expected to be reported for a study to be considered for use in a chemical assessment are generally also important for publishing research results in a way that ensures transparency and scientific credibility. The existing reporting requirements used in regulatory decision‐making did not evolve separated from science; they are the direct result of years of method development and research performed within the scientific community.

Therefore, peer‐reviewed research can provide information that is crucial for decision‐making to protect human health and the environment. However, to act as an adviser for society when decisions are to be taken, reporting of peer‐reviewed studies need to be thorough and accurate, thereby ensuring reproducibility and reliability of the published results. This can be achieved by improving reporting requirements for authors.

## CONFLICT OF INTEREST

The authors did not report any conflict of interest.

## References

[jat3578-bib-0001] Ågerstrand, M. , Berg, C. , Björlenius, B. , Breitholtz, M. , Brunström, B. , Fick, J. , … Rudén, C. (2015). Improving environmental risk assessment of human pharmaceuticals. Environmental Science & Technology, 49, 5336–5445. https://doi.org/10.1021/acs.est.5b00302. 25844810 10.1021/acs.est.5b00302

[jat3578-bib-0002] Ågerstrand, M. , Sobek, A. , Lilja, K. , Linderoth, M. , Wendt‐Rasch, L. , Wernersson, A.‐S. , & Rudén, C. (2017). An academic researcher's guide to increased impact on regulatory assessment of chemicals. Environmental Science: Processes & Impacts, 19, 644–655. https://doi.org/10.1039/C7EM00075H. 28452384 10.1039/c7em00075h

[jat3578-bib-0003] Alcock, R. E. , MacGillivray, B. H. , & Busby, J. S. (2011). Understanding the mismatch between the demands of risk assessment and practice of scientists – the case of deca‐BDE. Environment International, 37, 216–225. https://doi.org/10.1016/j.envint.2010.06.002. 20609476 10.1016/j.envint.2010.06.002

[jat3578-bib-0004] Beronius, A. , Ågerstrand, M. , Rudén, C. , & Hanberg, A. (2017). *SciRAP Workshop Report : Bridging the Gap between Academic Research and Chemicals Regulation – the SciRAP Tool for Evaluating Toxicity and Ecotoxicity Data for Risk Assessment of Chemicals* . Nordiska Ministerrådet. http://norden.diva-portal.org/smash/record.jsf?pid=diva2:1074866

[jat3578-bib-0005] Beronius, A. , Molander, L. , Rudén, C. , & Hanberg, A. (2014). Facilitating the use of non‐standard in vivo studies in health risk assessment of chemicals: a proposal to improve evaluation criteria and reporting. Journal of Applied Toxicology, 34, 607–617. https://doi.org/10.1002/jat.2991. 24481642 10.1002/jat.2991

[jat3578-bib-0006] Boone, M. D. , Bishop, C. A. , Boswell, L. A. , Brodman, R. D. , Burger, J. , Davidson, C. , … Weir, S. (2014). Pesticide regulation amid the influence of industry. BioScience, 64, 917–922. https://doi.org/10.1093/biosci/biu138.

[jat3578-bib-0007] Brazma, A. , Hingamp, P. , Quackenbush, J. , Sherlock, G. , Spellman, P. , Stoeckert, C. , … Vingron, M. (2001). Minimum Information about a Microarray Experiment (MIAME) – toward Standards for Microarray Data. Nature Genetics, 29, 365–371. https://doi.org/10.1038/ng1201-365. 11726920 10.1038/ng1201-365

[jat3578-bib-0008] European Chemicals Agency . (2011). REACH Guidance Documents. Helsinki, Finland. https://echa.europa.eu/guidance-Documents/guidance-on-Reach

[jat3578-bib-0009] Glasziou, P. , Altman, D. G. , Bossuyt, P. , Boutron, I. , Clarke, M. , Julious, S. , … Wager, E. (2014). Reducing waste from incomplete or unusable reports of biomedical research. The Lancet, 383, 267–276. https://doi.org/10.1016/S0140-6736(13)62228-X. 10.1016/S0140-6736(13)62228-X 24411647

[jat3578-bib-0010] Hanson, M. L. , Wolff, B. A. , Green, J. W. , Kivi, M. , Panter, G. H. , Warne, M. S. J. , … Sumpter, J. P. (2017). How we can make ecotoxicology more valuable to environmental protection. Science of the Total Environment, 578, 228–235. https://doi.org/10.1016/j.scitotenv.2016.07.160. 27503632 10.1016/j.scitotenv.2016.07.160

[jat3578-bib-0011] Hartmann, N. B. , Ågerstrand, M. , Lützhøft, H.‐C. H. , & Baun, A. (2017). NanoCRED: A transparent framework to assess the regulatory adequacy of ecotoxicity data for nanomaterials – relevance and reliability revisited. NanoImpact, 6, 81–89. https://doi.org/10.1016/j.impact.2017.03.004.

[jat3578-bib-0012] Kase, R. , Korkaric, M. , Werner, I. , & Ågerstrand, M. (2016). Criteria for Reporting and Evaluating Ecotoxicity Data (CRED): Comparison and Perception of the Klimisch and CRED methods for evaluating reliability and relevance of ecotoxicity studies. Environmental Sciences Europe, 28 https://doi.org/10.1186/s12302-016-0073-x. 10.1186/s12302-016-0073-x PMC5044958 27752442

[jat3578-bib-0013] Kilkenny, C. , Browne, W. J. , Cuthill, I. C. , Emerson, M. , & Altman, D. G. (2010). Improving bioscience research reporting: The ARRIVE Guidelines for Reporting Animal Research. PLoS Biology, 8, e1000412 https://doi.org/10.1371/journal.pbio.1000412. 20613859 10.1371/journal.pbio.1000412 PMC2893951

[jat3578-bib-0014] Kortenkamp, A. , Martin, O. , Faust, M. , Evans, R. , McKinlay, R. , Orton, F. , & Rosivatz, E. (2011). State of the Art Assessment of Endocrine Disrupters. Final Report. http://ec.europa.eu/environment/chemicals/endocrine/pdf/sota_edc_final_report.pdf 10.3109/10408444.2012.712943 22900988

[jat3578-bib-0015] Lillicrap, A. , Macken, A. , & Thomas, K. V. (2015). Recommendations for the inclusion of targeted testing to improve the regulatory environmental risk assessment of veterinary medicines used in aquaculture. Environment International, 85, 1–4. https://doi.org/10.1016/j.envint.2015.07.019. 26291502 10.1016/j.envint.2015.07.019

[jat3578-bib-0016] Moermond, C. T. , Kase, R. , Korkaric, M. , & Ågerstrand, M. (2016). CRED: Criteria for reporting and evaluating ecotoxicity data. Environmental Toxicology and Chemistry, 35, 1297–1309. https://doi.org/10.1002/etc.3259. 26399705 10.1002/etc.3259

[jat3578-bib-0017] Nature (2013). Announcement: Reducing our irreproducibility. Nature, 496, 398–398. https://doi.org/10.1038/496398a.

[jat3578-bib-0018] EFSA Scientific Committee (2013). Scientific Opinion on the hazard assessment of endocrine disruptors: Scientific criteria for identification of endocrine disruptors and appropriateness of existing test methods for assessing effects mediated by these substances on human health and the environment. EFSA Journal, 11, 3132 https://doi.org/10.2903/j.efsa.2013.3132.

[jat3578-bib-0019] Vandenbroucke, J. P. , von Elm, E. , Altman, D. G. , Gøtzsche, P. C. , Mulrow, C. D. , Pocock, S. J. , … for the STROBE Initiative (2007). Strengthening the Reporting of Observational Studies in Epidemiology (STROBE): Explanation and Elaboration. PLoS Medicine, 4, e297 https://doi.org/10.1371/journal.pmed.0040297. 17941715 10.1371/journal.pmed.0040297 PMC2020496

[jat3578-bib-0020] Zoeller, R. T. , Brown, T. R. , Doan, L. L. , Gore, A. C. , Skakkebaek, N. E. , Soto, A. M. , … Vom Saal, F. S. (2012). Endocrine‐disrupting chemicals and public health protection: A statement of principles from the Endocrine Society. Endocrinology, 153, 4097–4110. https://doi.org/10.1210/en.2012-1422. 22733974 10.1210/en.2012-1422 PMC3423612

